# Large-Scale Comparative Study Between Microendoscopic Laminectomy and Full-Endoscopic Laminectomy for the Treatment of Single-Level Lumbar Spinal Canal Stenosis

**DOI:** 10.7759/cureus.52842

**Published:** 2024-01-24

**Authors:** Kento Takebayashi, Muneyoshi Fujita, Takahiro Inui, Yasushi Oshima, Hiroki Iwai, Hirohiko Inanami, Hisashi Koga

**Affiliations:** 1 Neurosurgery, Iwai Full-Endoscopic Spine Surgery Clinic, Tokyo, JPN; 2 Orthopaedic Surgery, Teikyo University School of Medicine, Tokyo, JPN; 3 Orthopaedic Surgery, University of Tokyo, Tokyo, JPN; 4 Spine Surgery, Iwai Orthopaedic Hospital, Tokyo, JPN; 5 Orthopaedic Surgery, Inanami Spine and Joint Hospital, Tokyo, JPN; 6 Neurosurgery, Iwai Orthopaedic Medical Hospital, Tokyo, JPN

**Keywords:** treatment, minimally invasive, full-endoscopic laminectomy, microendoscopic laminectomy, lumbar spinal canal stenosis

## Abstract

Background: We previously compared the operative outcomes of microendoscopic laminectomy (MEL) and full-endoscopic laminectomy (FEL) for single-level lumbar spinal canal stenosis (LSCS). In this initial report, the operative outcomes of FEL were not inferior to those of MEL.

Objective: The purpose of this study is to compare the outcomes of MEL and FEL for single-level LSCS on a large scale using widely used multiple evaluation methods.

Methods: MEL was performed using a 16 mm tubular retractor and an endoscope, while FEL was performed using a 6.4 mm working channel endoscope. A retrospective study was performed on patients with LSCS treated with MEL (n = 355) or FEL (n = 154). Patient background and operative data were also collected. The Oswestry Disability Index (ODI), European Quality of Life-5 Dimensions (EQ-5D), and 36-item Short Form Survey (SF-36) scores were recorded preoperatively and 1-year postoperatively.

Results: Background data of the two groups and the mean operation time (MEL, 72.1 m; FEL, 74.2 m) were not significant (p>0.2). The mean volumes of intraoperative bleeding (MEL, 25.2 ml; FEL, 10.3 ml) were significantly different (p<0.001). The mean postoperative hospital stays (MEL, 3.9 days; FEL, 2.1 days) were significantly different (p<0.001). Fifteen dural tears (MEL, 11; FEL, 4) and 1 surgical site infection (MEL, 1; FEL, 0) were observed but not significant (p>0.5). Reoperation was required for postoperative hematoma in five patients (MEL, 3; FEL, 2). Although the ODI, EQ-5D, and SF-36 scores improved significantly at one year postoperatively in the MEL and FEL groups (p<0.001), there were no significant differences between the two groups (p>0.1).

Conclusion: The operative outcomes and minimal invasiveness were no statistical difference between the MEL and FEL groups. Further development of the operative techniques and the instruments of FEL are required to shorten the operation time.

## Introduction

Lumbar spinal canal stenosis (LSCS) is frequently observed in elderly people more than 65 years old, and the number of patients with LSCS has been increasing with an aging society. Prevention of postoperative delirium is an important issue in the surgical treatment of patients with LSCS who are of advanced age [1]. Therefore, considerable attention has been paid to minimally invasive treatments that can achieve early bed-leaving. Several strategies are available to treat LSCS. Microendoscopic laminectomy (MEL), which uses a 16 mm diameter tubular retractor and an endoscope, is an established minimally invasive treatment method for LSCS [2,3]. MEL is classified as an endoscope-assisted surgery according to the nomenclature proposed by AOSpine [4]. Although there are minor modifications to this approach (such as the paramedian and midline approaches), MEL is one of the standard operative procedures for LSCS in Japan and has been performed on nearly all LSCS in our hospital until May 2019.

In contrast, full-endoscopic laminectomy (FEL) is classified as a full-endoscopic spine surgery (FESS) performed under continuous saline irrigation during surgery [5-7]. Therefore, a clear visual field is provided by the washout of intraoperative bleeding. Furthermore, the light-collecting part of the endoscope is located at its tip. The intraoperative view can be maximized by placing the endoscope close to the target tissue. Previously, FEL used an approximately 4 mm working channel endoscope to treat foraminal and lateral recess stenoses [8-10]. Although treating central-type LSCS is possible using this type of small working channel endoscope, a wide range of laminectomies were time-consuming. Recently, a 6.4 mm working channel endoscope for FEL has become available in Japan [11,12]. We applied this system to treat LSCS in June 2019.

FEL seems to be more minimally invasive than MEL, not only for small skin incisions but also for reduced damage to surrounding structures such as muscles, facet joints, and interspinous ligaments. Currently, only a small number of comparative studies are available [9,11,13-19], and we therefore compared the operative outcomes between MEL and FEL for single-level LSCS in 2020 [11]. In this initial report, we only compared short-term operative outcomes for a small number of patients (MEL 54 cases, FEL 60 cases) using a postoperative NRS score obtained at leaving the hospital and a satisfaction score recorded at 2 points (leaving the hospital and three months after MEL or FEL). We originally established the satisfaction score and an eleven-level rating scale, similar to the NRS [20].

In this preliminary study, we showed no inferiority of FEL over a short follow-up period against MEL for treating single-level LSCS. We, therefore, designed a 1-year comparative study for a large number of patients using widely used evaluation scores for lumbar diseases to examine the generalizability of this feasibility study.

## Materials and methods

Study design

A retrospective comparative study was performed using the patients' data between Jun 2019 and March 2022. The inclusion criteria of this study were single-level LSCS who underwent laminectomy in our hospital. All patients had neurogenic intermittent claudication and/or apparent radiculopathy. Conserved therapies (medical treatment, epidural steroids, and/or nerve blocks) were performed for at least three months, and surgical treatment was only performed in unimproved cases. We excluded patients in whom we could not determine whether radiculopathy was caused by combined foraminal stenosis. Spondylolisthesis, severe instability assessed by gross motion (> 3 mm) on flexion-extension lumbar lateral radiographs, and severe degenerative scoliosis (coronal Cobb angle > 15°) were also excluded from the study [11]. METRx endoscopic system (Medtronic Sofamor Danek, Memphis, TN, USA) or a 6.4 mm working channel endoscope (TOKIBO CO., LTD, Tokyo, Japan) were used for the laminectomy. We finally analyzed a total of 509 (MEL, 355; FEL, 154) consecutive patients.

Data collection

Age, sex, height, weight, body mass index (BMI), and operated vertebral level were collected as patient background data from each operative record. Extension of the LSCS, and the target area for decompression were determined by preoperative computed tomography (CT) images and magnetic resonance images (MRI).

The operation time, intraoperative bleeding, postoperative hospital stay, and complications related to the operation were also extracted from the records. Intraoperative bleeding was calculated by subtracting the amount of irrigation saline from that of suction. The lowest amount obtained using this formula in both the MEL and FEL groups was 5 ml; we, therefore, determined that the measuring limit was 5 ml, and unmeasurable cases were recorded as 5 ml. Operative outcomes were evaluated using the preoperative and 1-year postoperative Oswestry Disability Index (ODI), European Quality of Life-5 Dimensions (EQ-5D), and 36-item Short Form Survey (SF-36) [Physical Functioning (PF), Bodily Pain (BP), Role-Physical (RP), Mental Health (MH)].

Data analysis

Data are presented as means and standard deviations (SD). The STATA version 16.0 (Stata Corp. LLC, College Station, TX, USA) was used for the following analyses: 1) t-test for continuous variables and chi-square test for categorical variables (comparison between demographic data and outcome measurements), 2) paired t-tests (comparison between preoperative and postoperative outcome measurements) [5-7]. P-values of <0.05 were considered to indicate significant differences.

Operative procedure

**Figure 1:**
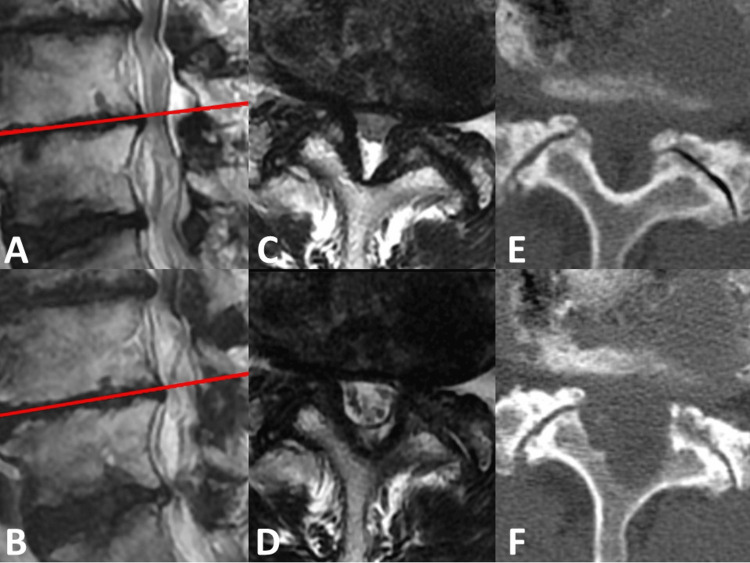
Bilateral decompression via unilateral approach (BDUA) performed by a 6.4-mm working channel FEL (A, B) Preoperative (A) and postoperative (B) sagittal T2-weighted magnetic resonance images (MRI) of the patient (78 years old, male) with L4/5 LSCS. The red lines indicate the scanning positions for axial MRI. (C, D) Preoperative (C) and postoperative (D) axial T2-weighted MRI. (E, F) Preoperative (E) and postoperative (F) axial computed tomography findings at the same scanning positions as MRI.

Both MEL and FEL surgeries were performed in a prone position under general anesthesia. The motor-evoked potential was monitored during the operative procedures to prevent nerve injury. For MEL, 10 skilled spinal surgeons who have experienced hundreds of spine surgery performed bilateral decompression via the unilateral approach (BDUA) or the midline approach (muscle-preserving interlaminar decompression). The basic operative procedure has been described previously by Baba and Oshima [21,22]. For FEL, a single skilled surgeon (H Koga) performed or assisted BDUA under fluoroscopic guidance (Figure 1). The detailed operative procedure has been described in our previous studies [11,12]. According to the pharmaceutical company's manual, antiplatelet drugs and anticoagulants were stopped during the perioperative term. Generally, drainage tubes were placed on all patients and pulled out the following day. All LSCS patients who visited Koga's outpatient clinic performed FEL. Other surgeons mainly performed MEL, and a few FEL were performed by other surgeons under Koga's assistance.

## Results

This retrospective study included 355 patients in the MEL group (224 men and 131 women) and 154 patients in the FEL group (94 men and 60 women) with single-level LSCS. The mean age at surgery was 68.8 years and 69.2 years in the MEL and FEL groups, respectively. The mean body height and weight were 161.6 cm and 63.0 kg in the MEL group and 162.7 cm and 63.0 kg in the FEL group, respectively. There were no significant differences in patient backgrounds between the MEL and FEL groups. We also compared the target vertebral levels and found no significant differences in the distribution between the MEL and FEL groups. Table 1 indicates patients' background data.

**Table 1:** Background data of 509 patients MEL: microendoscope laminectomy; FEL: full-endoscopic laminectomy; N: number; SD: standard deviation; BMI: Body Mass Index; ODI: Oswestry Disability Index; EQ-5D: European Quality of Life-5 Dimensions; SF-36: 36-item Short Form Survey; PF: Physical Functioning; BP: Bodily Pain; RP: Role-Physical; MH: Mental Health

Backgrounds	MEL (N=355)	FEL (N=154)	p-value
Age, mean (SD)	68.8 (10.5)	69.2 (10.5)	0.63
Sex (male) [N (%)]	224 (63.1)	94 (61.0)	0.66
Body height (cm), mean (SD)	161.6 (11.3)	162.7 (9.6)	0.27
Body weight (kg), mean (SD)	63.0 (12.1)	63.0 (11.1)	0.97
BMI	24.2 (6.5)	23.7 (2.8)	0.32
vertebral level [N (%)]			0.57
L1/2	3 (0.8)	1 (0.8)	
L2/3	22 (6.2)	7 (4.5)	
L3/4	63 (17.7)	36 (23.4)	
L4/5	247 (69.6)	102 (66.2)	
L5/L6	3 ( 0.8)	0 ( 0.0)	
L5/S1	17 ( 4.8)	8 ( 5.2)	
Preoperative ODI, mean (SD)	37.6 (13.7)	37.8 (15.3)	0.91
Preoperative EQ-5D, mean (SD)	0.60 (0.14)	0.60 (0.15)	0.91
Preoperative SF-36, mean (SD)			
PF	26.8 (15.7)	27.1 (16.1)	0.81
BP	31.5 (7.4)	31.4 (7.7)	0.90
RP	28.4 (14.3)	27.7 (13.0)	0.62
MH	49.2 (14.1)	48.2 (15.6)	0.49

The mean operation times of the MEL (72.1 min) and FEL groups (74.2 min) were not statistically different. The intraoperative bleeding in the FEL group (10.7 ml) was statistically lower than that in the MEL group (25.2 ml) (p < 0.001). A similar result was observed for postoperative hospital stay (MEL 3.9 days, FEL 2.1 days, p < 0.001) (Table 2).

Regarding complications, dural tears occurred in 11 patients in the MEL group, and dural sutures were required in two patients who suffered large dural tears (≧ 3mm) with cerebrospinal fluid (CSF) leakage. A human fibrinogen compound (KM Biologics Co. Ltd., Kumamoto, Japan) and a polyglycolic acid (GUNZE MEDICAL LIMITED, Osaka, Japan) sheet were used to repair small tears (< 3mm) in the remaining nine patients. Dural tears occurred in four patients in the FEL group, but the dural tear was not noticed during the operation, and a small amount of CSF leakage from the drainage tube was noticed after the operation in two patients. The dural tears of the remaining two patients were very small (<1mm), and the arachnoid membrane was intact. A polyglycolic acid sheet was used in one patient, and no further treatment was required for the dural tear. Surgical site infection was observed in one patient in the MEL group and required debridement under local anesthesia. Operative removal was required for postoperative hematoma in five patients (MEL, 3; FEL, 2) within six postoperative days. Evacuation in the FEL group was performed in the early stages of this study when the postoperative drainage tube was not placed. Following these accidents, we placed a drainage tube to prevent postoperative hematoma. The complication rates of the MEL and FEL groups were not statistically different (Table 2).

**Table 2:** Univariate analysis of operative outcomes for single-level LSCS LSCS: lumbar spinal canal stenosis; MEL: microendoscope laminectomy; FEL: full-endoscopic laminectomy; N: number; SD: standard deviation; SSI: surgical site infection; ODI: Oswestry Disability Index; EQ-5D: European Quality of Life-5 Dimensions; SF-36: 36-item Short Form Survey; PF: Physical Functioning; BP: Bodily Pain; RP: Role-Physical; MH: Mental Health

Operative outcomes	MEL (N=355)	FEL (N=154)	P value
Operation time (min), mean (SD)	72.1 (28.3)	74.2 (16.2)	0.45
Intraoperative bleeding (ml), mean (SD)	25.2 (42.6)	10.7 (8.3)	< 0.001
Postoperative Hospital Stay (days), mean (SD)	3.9 (1.3)	2.1 (0.6)	< 0.001
Complications			
Dural tear [N (%)]	11 ( 2.1)	4 (2.6)	0.76
SSI [N (%)]	1 ( 0.3)	0 (0)	0.51
Postoperative hematoma [N (%)]	3 ( 0.8)	2 (1.3)	0.63
ODI after 1-year, mean (SD)	17.0 (14.8)	17.8 (14.7)	0.60
EQ-5D after 1-year, mean (SD)	0.79 (0.19)	0.77 (0.18)	0.16
SF-36 after 1-year, mean (SD)			
PF	41.0 (15.0)	39.5 (15.2)	0.32
BP	45.4 (10.9)	43.7 (10.2)	0.10
RP	42.6 (13.6)	41.5 (13.1)	0.41
MH	61.0 (13.1)	59.1 (12.7)	0.14

The preoperative ODI and EQ-5D scores in the MEL group improved significantly 1-year postoperatively from 37.6 ± 13.7 and 0.60 ± 0.14 to 17.0 ± 14.8 and 0.79 ± 0.19, respectively (p < 0.001). The preoperative ODI and EQ-5D scores in the FEL group improved significantly postoperatively from 37.8 ± 15.3 and 0.60 ± 0.15 to 17.8 ± 14.7 and 0.77 ± 0.18, respectively (p < 0.001). The ODI and EQ-5D scores were not significantly different between the MEL and FEL groups, even at both observation points (Tables 1-3).

The preoperative SF-36 scores in the MEL group for PF, BP, RP, and MH improved significantly 1 year postoperatively from 26.8 ± 15.7, 31.5 ± 7.4, 28.4 ± 14.3, and 49.2 ± 14.1 to 41.0 ± 15.0, 45.4 ± 10.9, 42.6 ± 13.6, and 61.0 ± 13.1, respectively (p < 0.001). The preoperative SF-36 scores in the FEL group for PF, BP, RP, and MH improved significantly postoperatively from 27.1 ± 16.1, 31.4 ± 7.7, 27.7 ± 13.0, and 48.2 ± 15.6 to 39.5 ± 15.2, 43.7 ± 10.2, 41.5 ± 13.1, and 59.1 ± 12.7, respectively (p < 0.001). The SF-36 scores were not significantly different between the MEL and FEL groups, even at both observation points (Tables 1-3).

**Table 3:** Comparison between preoperative and postoperative ODI, EQ-5D, and SF-36 MEL: microendoscope laminectomy; FEL: full-endoscopic laminectomy; N: number; SD: standard deviation; SSI: surgical site infection; ODI: Oswestry Disability Index; EQ-5D: European Quality of Life-5 Dimensions; SF-36: 36-item Short Form Survey; PF: Physical Functioning; BP: Bodily Pain; RP: Role-Physical; MH: Mental Health

Operative procedures	Evaluation score	Preoperative	Postoperative	p-value
MEL (N=355)	ODI, mean (SD)	37.6 (13.7)	17.0 (14.8)	< 0.001
	EQ-5D, mean (SD)	0.60 (0.14)	0.79 (0.19)	< 0.001
	SF-36, mean (SD)			
	PF	26.8 (15.7)	41.0 (15.0)	< 0.001
	BP	31.5 (7.4)	45.4 (10.9)	< 0.001
	RP	28.4 (14.3)	42.6 (13.6)	< 0.001
	MH	49.2 (14.1)	61.0 (13.1)	<0.001
FEL (N=154)	ODI, mean (SD)	37.8 (15.3)	17.8 (14.7)	< 0.001
	EQ-5D, mean (SD)	0.60 (0.15)	0.77 (0.18)	< 0.001
	SF-36, mean (SD)			
	PF	27.1 (16.1)	39.5 (15.2)	< 0.001
	BP	31.4 (7.7)	43.7 (10.2)	< 0.001
	RP	27.7 (13.0)	41.5 (13.1)	< 0.001
	MH	48.2 (15.6)	59.1 (12.7)	<0.001

## Discussion

Before comparing the 1-year outcomes, we first confirmed that the patient background (age, sex, height, weight, and BMI) was not different between the two groups. Our analysis showed that FEL was superior in terms of shorter hospital stays and less intraoperative bleeding. Although the patients who received MEL were bed-rested until the following day as before, the patients who received FEL were permitted to walk in the ward 3 hours after the operation. As no problem was observed in this postoperative dealing with FEL, FEL seems to achieve early bed-leaving and subsequently shorter hospital stays.

Although we did not show a statistically significant difference in the operation time and complication rate of FEL versus MEL in this study, FEL might reduce the complication rate. All complications in the FEL group were observed only during the early stage of the learning curve. This included a stage in which the learning curve did not achieve a constant technical level. Particularly for dural tears, we established a technique to strip the adherent yellow ligament from the dura mater (supplementary video 1). The characteristics of the FESS endoscope (oblique-viewing type), in which the light-collection part of the endoscope is located at the tip, and the endoscope has a 15° oblique angle, make it possible to perform this technique. In addition, a wider working channel allows the use of a curette with a relatively thick handle to separate the adherent tissues from the dura mater. We detached the adhesion using a firm curette by closely observing the adherent region. Since developing and applying this technique, the patient has not suffered dural tears.

**Video 1:** The newly established technique to strip the adherent yellow ligament from dura mater The adherent yellow ligament was safely detached from the ventral dura mater by an originally developed curette with a small angled cap. The FESS endoscope (oblique-viewing type), in which the light-collection part of the endoscope is located at the tip and has a 15° oblique angle, makes it possible to perform this technique. FESS: full-endoscopic spine surgery

Another major issue is postoperative hematoma in both MEL and FEL. Drainage tubes were used for all MEL cases; however, postoperative hematoma occurred in three cases (0.8%). Several hemostatic materials, along with drainage tubes, were used during the surgical procedure in 70 patients who underwent MEL (19.7%). We used a drainage tube for the first 20 cases who underwent FEL. However, the volume of drainage was less than 5 ml, and therefore we temporarily stopped using a drainage tube. Subsequently, we experienced a patient with postoperative hematoma and started the use of a drainage tube again, following which we never again experienced a postoperative hematoma. Thus, we recommend using a drainage tube, even for FEL, which results in less intraoperative bleeding and reduced use of hemostatic materials (such materials were not used for any FEL in this study).

Surgical techniques for intraoperative hemostasis are also important to reduce intraoperative bleeding and postoperative hematoma. Bleeding from the bone surface, which is drilled using high-speed steel or diamond burrs, is the most troublesome. There are several basic hemostatic procedures: (I) low-speed drilling with a fine diamond burr, (II) electrocoagulation using a bipolar radio-frequency electrode system, (III) collapse of the bleeding cancellous bone using a Kerrison rongeur, and (IV) pasting of bone wax. Techniques I-III are essential in the field of FESS for the treatment of LSCS using a large working-channel endoscope [11,23,24].

Recently, Kotheeranurak et al. conducted a randomized controlled study of full-endoscopic (FE) and tubular-based microscopic (TM) decompression in 60 patients (FE, 30; TM, 30) with single-level LSCS [15]. Although they analyzed the ODI, visual analog scale, EQ-5D, walking time, and satisfaction rate (modified MacNab criteria) 24 months postoperatively, the FE group was non-inferior to the TM group. Mean blood loss was significantly lower in the FE group (p < 0.001), and the length of hospital stay after surgery was significantly shorter in the FE group (p = 0.011). These results are similar to those of our study, except for the complication rates (10% in the FE group and 20% in the TM group), which were higher than those in our study.

Chen et al. reported a retrospective study comparing endoscopic and microscopic BDUA in 93 patients (endoscopic, 42; microscopic, 51) with L4/5 LSCS [25]. Although there were no significant differences in leg pain and ODI, the endoscopic BDUA group had a significantly lower visual analog scale score for back pain, less analgesic use (p < 0.05), and lower mean blood loss and length of hospital stay after surgery. These results also support our outcomes. We further measured the improvement rates of ODI, EQ-5D, and SF-36 scores at each vertebral level. The improvement rates in ODI, EQ-5D, and SF-36 scores at 1 year after MEL and FEL were not significantly different in each vertebral level (data not shown) and not just for L4/5 LSCS (MEL 247, FEL 102).

Study limitations

Our study was not prospective, was not randomized, and was not a multi-institutional study, which is a limitation. However, the patient backgrounds (age, sex, body height, body weight, and BMI) and vertebral level of LSCS between the MEL and FEL groups were matched to the best of our experiences, and the analysis was concentrated on single-level central LSCS. There was selection bias in operative procedures depending on surgeons, which is another limitation. Furthermore, longer follow-up periods are required to evaluate long-term outcomes and complications.

## Conclusions

In this retrospective study, we showed that the operative outcomes (1-year follow-up period) of MEL and a 6.4 mm working channel FEL for single-level LSCS were the same. In addition to less intraoperative bleeding, FEL has an advantage in early bed-leaving. Furthermore, FEL achieved a reduction in using hemostatic materials. Although several surgical techniques have already been established to prevent surgical complications for FEL, the development of new surgical instruments for FEL is also expected for enhancement.
